# Function and Interactions of ERCC1-XPF in DNA Damage Response

**DOI:** 10.3390/molecules23123205

**Published:** 2018-12-05

**Authors:** Maryam Faridounnia, Gert E. Folkers, Rolf Boelens

**Affiliations:** Bijvoet Center for Biomolecular Research, Utrecht University, Padualaan 8, 3584 CH Utrecht, The Netherlands; m.faridounnia@gmail.com (M.F.); g.e.folkers@uu.nl (G.E.F.)

**Keywords:** ERCC1, XPF, DSB repair, ICL repair, NER, DNA damage response, Fanconi anemia

## Abstract

Numerous proteins are involved in the multiple pathways of the DNA damage response network and play a key role to protect the genome from the wide variety of damages that can occur to DNA. An example of this is the structure-specific endonuclease ERCC1-XPF. This heterodimeric complex is in particular involved in nucleotide excision repair (NER), but also in double strand break repair and interstrand cross-link repair pathways. Here we review the function of ERCC1-XPF in various DNA repair pathways and discuss human disorders associated with ERCC1-XPF deficiency. We also overview our molecular and structural understanding of XPF-ERCC1.

## 1. Introduction

Conservation and stability of genetic information not only relies on an accurate mechanism for copying DNA sequences before cell division, but also on the accurate maintenance of the genomic information by the DNA repair machinery [1,2,3,4]. Numerous damaging agents endanger the DNA: a major group of factors responsible for DNA lesions consists of external threats, such as those caused by ultraviolet (UV) light, ionizing radiation and genotoxic chemical agents. Another source of DNA lesions can be from the byproducts of metabolic activity, such as reactive oxygen species (ROS) that, if not removed by an antioxidant defense system, will cause oxidative modifications in DNA. Spontaneous or induced base deamination also cause lesions to the DNA. These are only a few examples of the many possible modifications that may occur in DNA continuously and which cause thousands of DNA lesions in each cell. Without a functional DNA damage response (DDR), when these damages remain uncorrected, these lesions would alter the DNA sequence so rapidly that it would be fatal for the organism: cells would cease to be viable or become malignant. The first response of the cell is a direct defense against these damaging agents through detoxification mechanisms and oxidative stress response. The biological response to DNA damage occurs through massive posttranslational modification by ATM and ATR protein kinases or protein kinase C leading to cell cycle arrest to enable DNA repair. If the damage is too severe, this can lead to senescence or apoptosis [1,2,3,4].

Depending on the type of damage, different DNA repair mechanisms have evolved to avoid the accumulation of DNA defects and to preserve the genetic information. The repair instruments range from single protein systems to multistep DNA repair pathways depending on the type of the lesion. Repair mechanisms are involved in either the reversal of DNA damage (e.g., photoreactivation [5,6]) or the excision of damaged components. This excision of damaged bases can be achieved either as free bases in base excision repair (BER), or as complete nucleotides in nucleotide excision repair (NER). Furthermore, mispaired bases can be repaired through mismatch repair and/or tolerated through translesional synthesis (TLS). In addition to damaged nucleotides, fractures in DNA threaten the integrity of genome which can be treated by double-strand break (DSB) or single-strand break (SSB) DNA repair mechanisms. Given the importance of the genome integrity, it is evident that impairments in DNA repair processes in human can lead to major genomic damage resulting for instance in inherited cancer predisposition syndromes, neurological disease, and premature aging [1,6,7,8,9].

The different DNA repair pathways share several steps during the DNA repair process and sometimes even the same factors are found in the different pathways. This is clearly the case for the structure-specific endonuclease ERCC1-XPF [6,10]. We will first overview the function of ERCC1-XPF in different DNA repair pathways, next review the physiological consequences of ERCC1-XPF deficiency and finally discuss the molecular and structural understanding of this protein complex.

## 2. Function of ERCC1-XPF in DNA Repair Pathways

The heterodimeric ERCC1-XPF complex is involved in a multitude of mechanisms and is known for its function among others in nucleotide excision repair (NER) (see also [11] for a recent review). The NER pathway is one of the most extensively studied DNA repair processes. It is characterized by cyclobutane pyrimidine dimer (CPDs) damages that are formed by exposing the DNA to UV irradiation. The role of the different proteins involved in this pathway, such as ERCC1-XPF, is well known. A multitude of discoveries indicate that ERCC1-XPF further functions in several other DNA repair pathways, to repair for instance SSBs, DSBs, and interstrand cross-links (ICLs), which might be due to its unique catalytic incision properties [6,12].

### 2.1. Nucleotide Excision Repair (NER)

**Figure 1:**
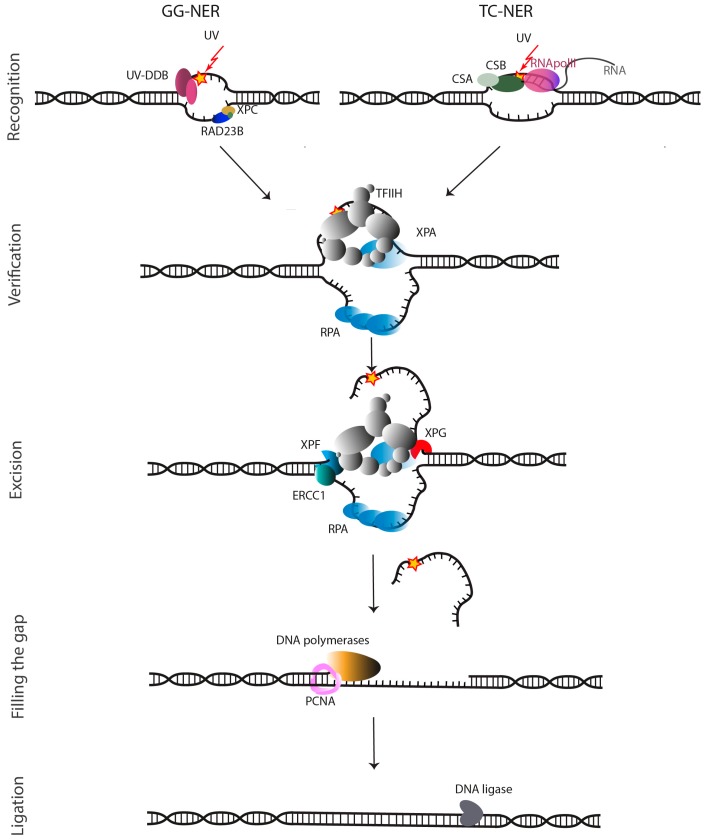
Schematic overview of the Nucleotide Excision Repair (NER) pathway. Lesion recognition leads to GG-NER, whereas stalled transcription leads to TC-NER. This is followed by damage verification, incision at 5′ side by ERCC1-XPF and 3′ side of the lesion by XPG. The gap left after the incisions is refilled by DNA re-synthesis. Adapted from [22,24,28].

In NER, complete nucleotides are removed from the damaged genome by a multiprotein repair process. These NER proteins perform a highly coordinated excision of the damage as a single-stranded oligonucleotide and restoration of the original DNA sequence using the non-damaged strand as a template [13,14]. The damages that are recognized by NER, have in common that they cause local bulky distortions in the DNA double helix. The DNA adducts—such as from oxidative DNA damage, thymidine and other CPDs, intra-strand cross-links, and bulky alkylating adducts induced by chemotherapy [4,15,16,17]—can be removed by two distinct subpathways: the global genome NER (GG-NER) and the transcription-coupled NER (TC-NER). In GG-NER, the entire genome is examined and upon recognition, oligonucleotide excision is initiated by lesion sensing DNA damage-binding proteins (DDBs) and the XPC-Rad23B complex [18,19,20]. On the other hand, in TC-NER RNA Polymerase II that is stalled at a DNA lesion, triggers recognition allowing to preferentially repair the transcribed strand of genes (Figure 1). When stalled at the lesion, the Cockayne syndrome proteins CSB and CSA translocate RNA Pol II, so that the DNA lesion becomes available for repair [21,22,23,24]. However, the mechanism is not fully understood [25,26,27].

After damage recognition, the two sub-pathways go through the same key events: formation of a pre-incision complex, followed by unwinding and excision of single-stranded DNA (ssDNA) nearby the lesion, gap filling, and sealing. An assembly of proteins, including transcription factor II H (TFIIH) and XPG, initiates the NER process [7]. Subsequently, the XPB subunit of TFIIH binds to the recognized damage site and through its ATPase activity enhances the binding of TFIIH to the damaged DNA [29,30]. Subsequently the helicase activity of the TFIIH XPD subunit that unwinds the damaged DNA into a so-called repair bubble. An open ssDNA structure is formed around the lesion consisting of typically 25–30 unpaired DNA bases containing the lesion. This structure is stabilized by the DNA-binding proteins RPA and XPA. ERCC1-XPF binds to DNA more efficiently in the presence of the replication protein A (RPA). RPA is believed to bind the non-damaged strand of DNA and to help to position the two endonucleases ERCC1-XPF and XPG and to protect the gap during the repair synthesis. The RPA binding site has been mapped to the N-terminus of XPF, and upon mutation of P85 to serine, XPF is unable to interact with RPA (Figure 1) [31,32].

XPA interacts with TFIIH, RPA, and DNA in the pre-incision complex, and then recruits ERCC1-XPF through its interaction with ERCC1 [33,34]. In addition to a core folded region, XPA has unstructured regions. One of these, encompassing a GGGF motif undergoes a disorder to order transition upon binding to the central domain of ERCC1 and is necessary and sufficient for the interaction with XPA and recruitment of ERCC1-XPF to damaged sites in DNA [35,36,37]. This stabilized repair bubble is the substrate for the dual incision activity of the two NER endonucleases, the ERCC1-XPF complex at the 5′ side and XPG at 3′ side, excising the damage containing 25–30 nucleotide single-stranded fragment. Previously thought to be a simple matter of cutting and pasting, dual incision and repair synthesis are now believed to be a complex and tightly coordinated process starting with 5′ incision by ERCC1-XPF, followed by initiation of repair synthesis, 3′ incision by XPG and completion of repair synthesis and ligation [38]. The repair synthesis involves at least three polymerases (Pol δ, Pol ε and Pol κ) and factors that use the intact DNA as template strand (Figure 1) [39]. Finally, the remaining nick is repaired by a DNA ligase [3,7,22,24,29,30].

A role of ERCC1-XPF in NER was first identified for its homologs in *S. cerevisiae.* RAD1 and RAD10 mutants that demonstrate hypersensitivity to UV irradiation, are defective in the incision step of NER [40,41]. Rad1-Rad10 was only functional as an endonuclease when present as a heterodimer. It showed specificity for substrates containing ss/dsDNA junctions with 3′ single-stranded overhangs, leading to a release of the 3′ overhangs [41,42]. Similarly, in higher eukaryotes, the roles of ERCC1 and XPF in NER was discovered by the ability of these proteins to restore UV resistance in NER-deficient Chinese Hamster Ovary (CHO) cell lines [43,44]. Subsequently, ERCC1-XPF was shown to possess structural specificity for ss/dsDNA junctions similar to Rad1-Rad10, consistent with the incision occurring 5′ to a lesion in NER [45,46].

### 2.2. Double-Strand Break Repair

Double-strand breaks (DSBs), are among the most deleterious types of DNA lesions, that must be repaired to maintain genome stability. DSBs can arise exogenously from ionizing radiation and chemical agents, or endogenously from errors from DNA replication and recombination, and cellular metabolism [47]. To avoid fragmentation, translocation and deletion of chromosomal DNA caused by DSBs cells have developed several repair pathways (Figure 1). 

DSBs are primarily repaired by homologous recombination (HR) and non-homologous end joining (NHEJ) being the major ones. If these two pathways fail to repair the damage, either single-strand annealing or an alternative NHEJ (Alt-NHEJ) pathway known as microhomology (MMEJ) can repair such damage, but with lower efficiency [48,49,50,51].

#### 2.2.1. Homologous Recombination (HR)

**Figure 2:**
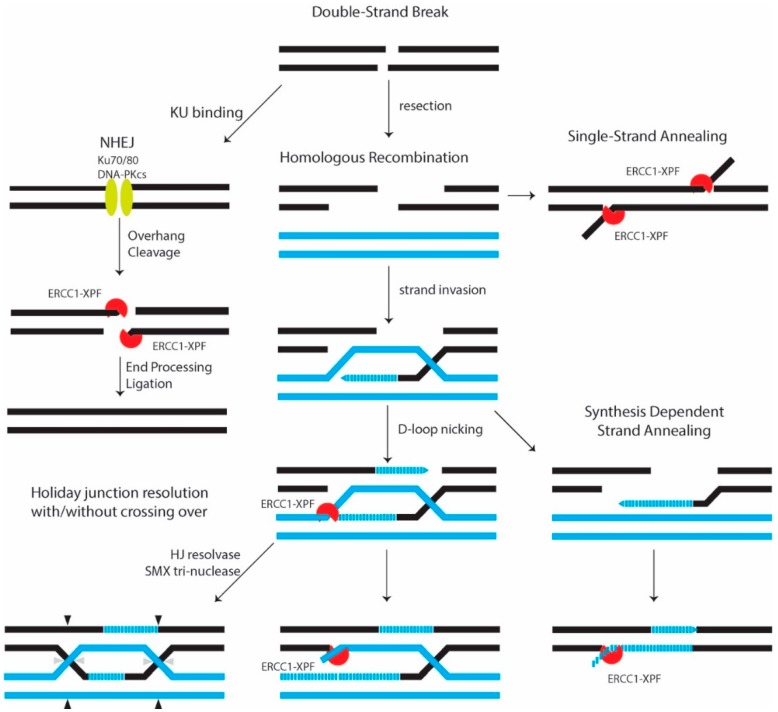
An overview of eukaryotic DSB repair. DSBs in DNA can be resealed through NHEJ (left) without homology requirement. ERCC1-XPF might trim the ends before resealing. Alternatively, DSBs repair can receive 5′ → 3′ single strand resection followed by invasion, which uses another homologous chromatid as template. This is followed either by re-annealing of the invading strands (synthesis-dependent strand annealing) or formation of a heteroduplex Holliday Junction. After a branch migration on this formed junction, DNA polymerase extends the missing sequence (D-loop formation). Also shown is how ERCC1-XPF may be involved at the different mechanisms of DSB repair. DNA copies from two distinct homologous chromatids are represented in blue and black. Adapted from [6,61,62,63].

HR repairs DSBs generated from DNA damage in late S/G2 and meiosis. After DSB resection, unidirectional 5′- to 3′-degradation of such a breakage results in single-stranded DNA overhangs. These 3′-overhangs are then coated by replication protein A (RPA) and with the aid of the BRCA2 protein, also known as Fanconi anemia protein FANCD1, multiple RAD51 proteins are targeted to the correct location on ssDNA. This process is known as filament formation [49,52,53,54,55]. During this process, ssDNA is prepared to invade a homologous DNA helix that can either be an available sister chromatid or homologous donor DNA sequences in the late S/G2 phase. This strand invasion recruits regions of undamaged DNA to form a template to restore the lost information [49,55,56]. Then RAD54 uses the filament for branch migration on the formed pairing intermediates (heteroduplex Holliday junction or D-loop) [49,57,58,59,60]. This continues with DNA polymerase extending the missing sequence. Resolving these Holliday junction intermediates can result in either crossovers or non-crossovers products. It has been suggested that an assembly of the SLX4-SLX1, MUS81-EME1, and ERCC1-XPF structure-specific endonucleases, named the SMX DNA-repair Tri-nuclease, can remove these potentially problematic branched DNA structures [61]. Other aspects of the repair process may proceed through different sub-pathways like single-strand annealing (SSA) mechanism (Figure 2) [49,56,57,58,59,62]. In recent years, the role of ERCC1-XPF in DSB repair has been gradually unraveled. For example, it is now clear that during single-strand annealing, ERCC1-XPF is responsible for the incision of the DNA [51,63]. Furthermore, XPF can interact with the *N*-terminal DNA-binding region of Rad52, in a DNA-independent manner. This interaction can promote the cleavage of 3′-overhangs by ERCC1-XPF and the processing of recombination intermediates that have arisen during the repair of DSBs [64,65].

#### 2.2.2. Non-Homologous End Joining (NHEJ)

NHEJ is another major mechanism in DSB repair that is activated during G0/G1 and early S-phases and is an end-joining mechanism that directly ligates the two ends in DNA strand breaks. This process that is not error-free process occurs before replication when another copy of DNA is absent. This process does not require any homologous sequence. Therefore, it utilizes a machinery that is distinct from those used in HR. The Ku70-Ku80 dsDNA-binding heterodimer (KU-DNA) recruits DNA-dependent protein kinase (DNA-PK) to the damaged region of the DNA, triggering complex formation. Since in most cases the endogenously generated DSBs do not have 3′ hydroxyl or 5′ phosphate overhangs, they lack essential molecular elements for polymerization and ligation. Therefore, the DSB ends have to be processed first, which includes the removal of the inadequacies, followed by polymerization and ligation. A recent model shows how after ligation, APLF (aprataxin and polynucleotide kinase-like factor) promotes the sealing of the repaired break [6,49,66,67,68,69,70,71]. It has been suggested that ERCC1-XPF is involved in NHEJ when DSB ends have extensive 3′-overhangs that need to be trimmed, and that this is regulated by DNA-dependent protein kinase (DNA-PK) within the context of the NHEJ complex (Figure 2) [51,72,73,74]. However, functional assays on XPF knock-out cell lines by Lehman et al. put this model in question and calls for revisiting the function of ERCC1-XPF in NHEJ [75].

#### 2.2.3. Microhomology Mediated End-Joining (MMEJ) 

MMEJ is an alternative NHEJ mechanism that occurs during S phase. During MMEJ short homologous DNA fragments of 5–20 nucleotides are joined in a rather error-prone manner, often yielding deletions, and leading to genome instability. Many questions remain unresolved about MMEJ. For example, a study by Deng et al. [76] shows that the protein RPA regulates this pathway. However, it is not clear whether MMEJ competes with HR components located on the RPA coated DNA, or if there is an alternative mechanism. One mechanistic model, proposed for MMEJ, starts with end resection, followed by SSA and DNA end processing, and finalized by ligation [68]. Before ligation, during the end processing, ERCC1-XPF plays a key role in the cleavage of non-complementary 3′-flaps from an annealed intermediate. This excision activity facilitates stable DNA end joining, proper gap filling and ligation [49,51,68,77].

### 2.3. Single-Strand Break Repair

Single-strand breaks (SSB) are one of the most common DNA damages that arise as intermediates of Base Excision Repair (BER), or directly from damage of the DNA deoxyribose by reactive oxygen species (ROS) or by inhibited activity of topoisomerase-1 (TOP1-SSB). Direct SSB repair of ROS damaged DNA is initiated by recognition and signaling performed by poly-ADP-ribose polymerases (PARP) 1 and 2, while SSBs introduced during BER or by TOP1 activity might require other recognition mechanisms. The PARP proteins, which bind to SSBs and signal their presence, are thought to attract XRCC1. This protein orchestrates the repair of SSBs by forming a scaffold for a number of enzymes responsible for processing and gap filling of DNA breaks [6,78,79,80,81].

Since the 3′- or 5′-terminus of many SSBs are distorted, they need end processing to meet the substrate requirements for gap filling and ligation. These distortions can be very diverse, and depending on the type, a repertoire of enzymes is required, including endonucleases that are acquired from different molecular pathways [6,78,79]. Related to this, it has been suggested that some of the 3′-termini are removed by ERCC1-XPF [79,82]. Most SSBs gap filling involves insertion of a single nucleotide, followed by XRCC1-dependent ligation. In some cases, when the incision is involved in end processing, gap filling involves insertion of more nucleotides [78,79,80].

### 2.4. Interstrand Cross-Link (ICL) Repair

**Figure 3:**
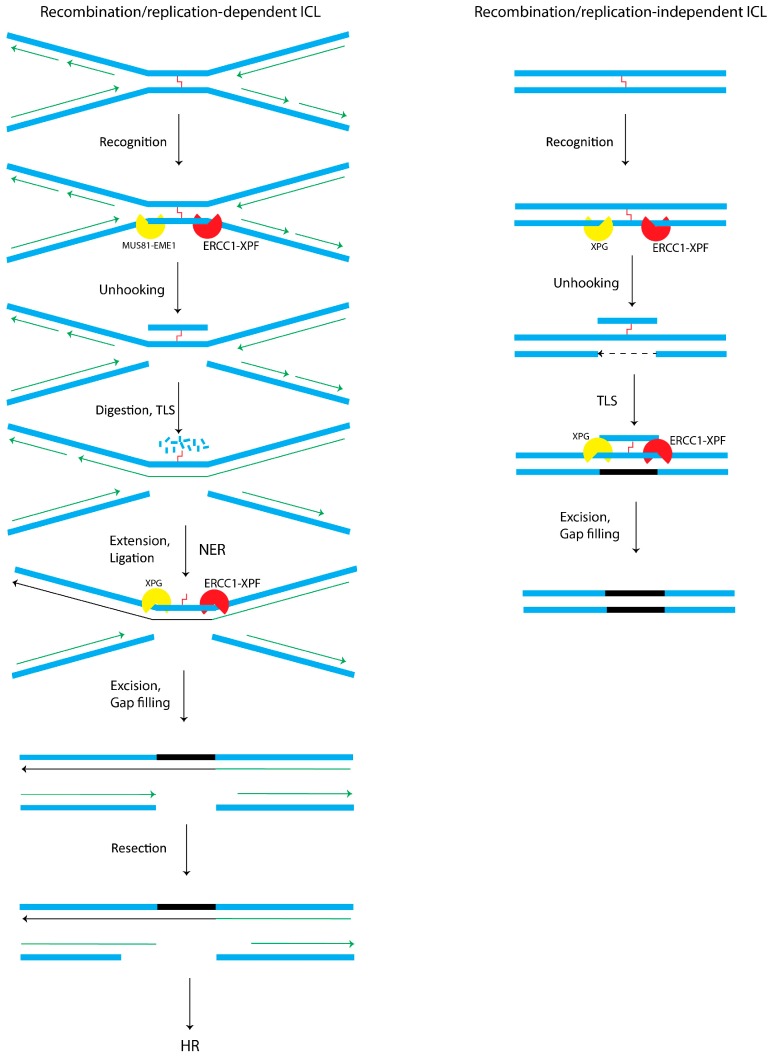
The mechanisms of ICL repair. ICLs cause the replication machinery to collapse at the replication fork (left panel) and create DSBs that cannot be repaired without excising and unhooking at both sides of ICLs, by Mus81-Eme1 and ERCC1-XPF, creating a one-sided DSB. This makes the strand accessible for TLS polymerases that bypass the unhooked crosslink in a mutagenic manner and the final lesion is removed via NER. The created two-sided DSB in the next strand is repaired through homologous recombination (HR). In the right panel, ICLs recognition occurs by stalled RNA polymerase during transcription, initiating TC-NER and ERCC1-XPF function. After the function of TLS, NER factors perform excision on the two sides of ICL, followed by gap filling and continuation of transcription. Adapted from [84].

Cytotoxic and antitumor agents can form covalent linkages on DNA in different ways including intra-strand, between two complementary strands, and even between a DNA base and a protein residue. ICLs, though accountable for only a small amount of the total DNA adducts, are one of the most deleterious DNA lesions because this covalent binding prevents DNA from separation and stalls replication and transcription processes, thus causing chromosome rearrangement or break [83,84]. These lesions arise either exogenously from exposure to toxic mutagens such as cisplatin and carcinogens in cigarette smoke, or endogenously from amino-acid metabolism or lipid peroxidation for instance from a high fat diet. The formed DNA adducts arrest replication and transcription. Using an ERCC1 deficient mouse model, it has been demonstrated that cross-linking agents such as 4-hydroxy-2-nonenal (HNE) do not only produce ICLs but can also cause multiple other DNA lesions that depending on damage type, may trigger a separate repair pathway [84,85]. ICL repair occurs during the S or G0/G1 phase, in the presence or absence of replication proteins. Therefore, ICL repair pathways are classified as replication-dependent and replication-independent (Figure 3) [83,84,86,87,88]. In both ICL repair processes, it is thought that the Fanconi anemia (FA) network coordinates the function of major groups of proteins from the NER pathway, the homologous recombination (HR) and the DNA translesion synthesis (TLS) pathway. Indeed, mutations in genes regulating the elimination of ICLs can cause FA [83,89]. It is increasingly understood that in ICL repair, FA proteins function through sensing, recognition and processing of ICLs [90].

#### 2.4.1. ICL Replication/Recombination-Dependent Pathway

The ICL replication/recombination-dependent pathway is initiated during the late S or G2 phase by a stalled replication fork at the ICL site. In general, the presence of an undamaged sister chromatid initiates the assembly of the HR machinery including RAD54, FA proteins, RPA and ERCC1-XPF complex (Figure 3) [83,91,92]. ICL damage triggers the FA pathway by stimulating ubiquitylation of FA proteins [93,94]. This activity is essential for SLX4 loading on this site [95]. SLX4 functions as a molecular scaffold for the assembly of proteins, ensures the participation of the nucleases MUS81-EME1 and ERCC1-XPF in the incision of DNA flanking the damage site, creates DSBs and unhooks the cross-link at the stalled replication fork. ERCC1-XPF interact with SLX4 to unhook ICLs.

The unhooked fragment is still linked to the template strand through its ICL [96,97,98,99,100,101]. At its *N*-terminal part, SLX4 interacts with ERCC1-XPF, but the precise binding regions have not been identified [102]. Using a mouse model, it is shown that SLX4 functions in ICL repair and that the *N*-terminal part of SLX4 is sufficient to confer resistance to DNA cross-linking agents. The complex formation between SLX4 and ERCC1-XPF enhances the ERCC1-XPF nuclease potency and shifts its DNA binding affinity towards DNA flaps with 3′-overhang and replication like structures [103,104]. It is believed that FANCD2 ubiquitination assists the trans-lesional polymerases in DNA synthesis at the lesion site, which is followed by the removal of reminiscent DNA flaps by the NER components ERCC1-XPF and XPG. This fully repaired sister chromatid is used for the repair of DSB intermediates through HR (Figure 2) [84,86,105,106,107]. Impairments in ICL repair can lead to FA disorder, characterized by chromosomal frailty, congenital abnormalities, bone marrow failure, increased cancer rate, and ICL hypersensitivity [84,86,87,88]. Little is known on the ubiquitin signaling in the NER pathway [108,109,110]. That the level of XPC protein is controlled by the ubiquitin-proteasome system, has been well established [108,111], but for other factors such as ERCC1-XPF this is less clear. It has been shown that the ERCC1-XPF complex can be ubiquitylated via the C-terminal (HhH)_2_ domains of ERCC1 [112]. Another study [113] demonstrated a direct interaction between the deubiquitylase USP45 and ERCC1, which may control its ubiquitylation. However, further studies are needed to determine the precise role of ubiquitination of ERCC1-XPF in the regulation of NER.

#### 2.4.2. ICL Replication-Independent Repair Pathway

The ICL replication-independent repair (RIR) pathway occurs during the G0/G1 phase and is vital for cellular homeostasis especially in post-mitotic cells, such as in neurons or in cells dividing rarely. RIR depends on NER for damage recognition and dual incision utilizing its endonucleases ERCC1-XPF and XPG to unhook the ICL lesion and to excise the DNA ICL lesion. This is followed by repair synthesis of the gap, performed by TLS polymerase activity (Figure 3) [83,99,114].

#### 2.4.3. Other ICL Repair Pathways

**Figure 4:**
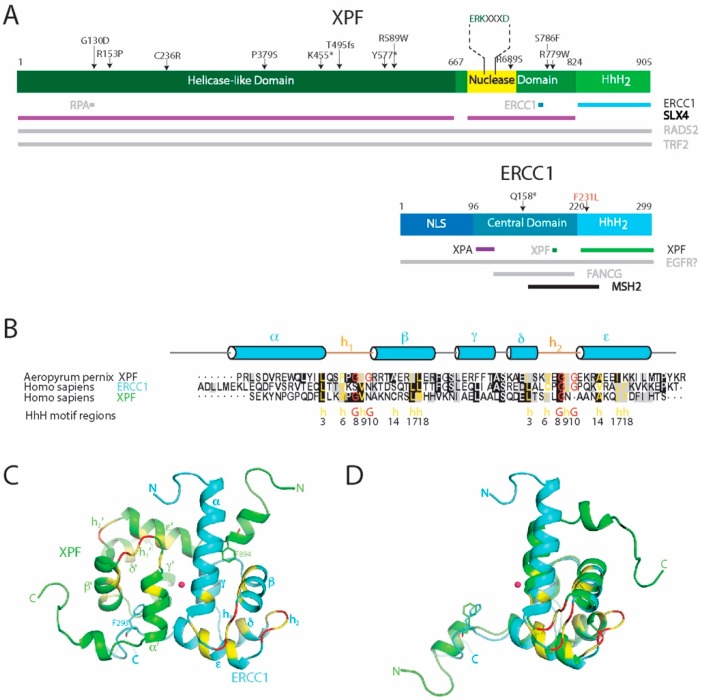
Schematic representation of human ERCC1-XPF. ERCC1 and XPF do not only interact with each other, but also with other proteins. **A**, shows the domain architecture, mutations and interactions. The expansion of key residues is represented above the active site (in yellow) within the XPF nuclease domain. Protein names in grey denote unconfirmed or undefined protein–protein interactions and confirmed interactions are written black. NLS is the abbreviation for nuclear localization sequence. Numbers denote residue number, arrows show mutations, asterisks are used to denote stop codons, and the horizontal bars identify the proteins. **B**, (HhH)_2_ domains of *A. pernix* XPF and human ERCC1 and XPF are compared by structure-based alignment using the DaliLite server [157] with sequence alignment colored in boxshade [grey for similar and black for identical [158]]. The signature residues based on multiple sequence alignment of HhH motifs by Doherty et al. [159] are manually colored (yellow for hydrophobic and red for Glycine) and denoted below the sequence (G indicates conserved glycine residues; h indicates a hydrophobic side chain). The secondary structure elements are shown based on the structure of human ERCC1 (HhH)_2_ domain. **C**, the NMR structure of heterodimeric complex of the human ERCC1-XFP (HhH)_2_ domains with the two-fold pseudo-symmetry axis (central red dot). **D**, Overlay of ERCC1 and XPF (HhH)_2_ structures using DaliLite [157], showing the overall fold similarity and close overlap of conserved F293 and F894 anchor residues. ERCC1 is depicted in blue, XPF in green, and the HhH motif signature resides are in yellow and red; GhG denotes signatures residues for hairpins. **A** and **B** and are adapted from [104] and [160], respectively.

Other functions described for ERCC1-XPF in ICL repair require more investigation. It is demonstrated that FANCG-deficient cells are not able to incise DNA at ICLs and that FANCG can also interact with the central domain in ERCC1 [87]. The FANCG binding within ERCC1 which involves residues 96–119 of cERCC1 [87] overlaps with the XPA binding site which involves residues 92–119 (cf. Figure 4). Mutagenesis studies map the interaction to the tetratricopeptide repeats TPR-1, TRP3, TRP-5, and TRP6 in FANCG. It is believed that this interaction plays a crucial role for recruiting ERCC1-XPF to ICLs, analogous to the recruitment of ERCC1-XPF to sites of damage in NER by XPA, but further studies are needed to elucidate the relevance of the interaction for crosslink repair [87,105].

Also, it has been suggested that the MutS homolog protein MSH2, a mismatch repair protein, is involved in the cellular response in cisplatin treated cells, due to its preferential binding to cisplatin ICLs containing a mismatch [115,116,117]. Since ERCC1-XPF is also involved in cisplatin resistance it has been suggested that there might exist an interaction between these proteins. Lan et al. reported that during an XPA-independent repair mechanism the ERCC1 C-terminal residues 184–260, might be involved in an interaction with MSH2 [115,118], suggesting a role for ERCC1 in MMR.

### 2.5. Roles of ERCC1-XPF Beyond DNA Repair

ERCC1-XPF heterodimer plays a key role in multiple DNA repair pathways. Nevertheless, not all phenotypes of ERCC1-XPF deficiencies can be interpreted by their primary function in a DNA repair pathway. Many reports associate unexplained phenotypes of ERCC1-XPF, such as developmental disorders and neurodegeneration, with a variety of activities beyond DNA repair. For instance, the ERCC1-XPF can also be involved in telomere maintenance and functions through direct or indirect interaction with the telomere repeats binding protein TRF2, independent of TRF2’s DNA binding activity [119,120]. In telomeres, DNA loop conformations are formed in long G-rich 3′-overhangs at the ends of chromosomes. These long ssDNA circles are stabilized by the shelterin protein, TRF2. ERCC1-XPF associates with TRF2 at these sites and its activity on 3′-overhangs depends on the presence of TRF2 [120]. So far, no direct interaction has been reported between TRF2 and ERCC1-XPF. Since also TRF2 interacts with SLX4, it is possible that the effects attributed to ERCC1-XPF are mediated by SLX4 in a ternary complex of XPF, SLX4 and TRF2 [11,121,122]. The XPF function in TRF2-mediated telomere shortening may not occur through its nuclease activity, since mutations in the conserved nuclease domain have no influence on telomere processing [123]. Though these studies on the regulation of DNA repair pathways and interactions at telomeres, particularly ERCC1-XPF and TRF2 interactions, are insightful, they are nevertheless scattered and speculative, and demand detailed molecular and biochemical engaged research.

Another example of ERCC1-XPF function outside DNA repair is its participation in sister chromatid separation during chromosome segregation. The ERCC1-XPF complex, together with MUS81-EME1, can be co-localized by FANCD2 to mitotic chromosomes and it is demonstrated that ERCC1 down-regulation causes defects in chromosome segregation, hence, mitotic failure [97,124].

It has been suggested that during transcription initiation ERCC1-XPF may fine-tune optimal transactivation of target genes that can affect cell development [125]. Recent evidence shows that ERCC1-XPF cooperates with the transcriptional inhibitor CTCF (CCCTC-binding factor) and the cohesion subunits SMC1A and SMC3 to facilitate gene silencing at the promoters and imprinted genes control regions (ICRs) during hepatic postnatal development [126]. Lack of ERCC1 or persistent DNA damage causes aberrant developmental expression of imprinted genes and chromatin changes that affect gene expression programs associated with NER disorder [126,127].

## 3. Physiological Consequences of Impaired ERCC1-XPF

In recent years, the role of ERCC1-XPF in other pathways has been understood through syndromes that are beyond symptoms of NER deficiency [11,88]. The manifestation and severity of ERCC1-XPF deficiencies are significantly determined by the role impaired due to the mutant alleles [7,128,129].

### 3.1. Xeroderma Pigmentosum (XP)

One of the most prominent diseases caused by NER defects, Xeroderma pigmentosum (XP), is an inborn disorder conferring dermal damage to sunlight-exposed skin areas. XP has been related to defective protection against UV damage, which results in a cancer-prone skin. XP patients also have an elevated frequency of internal cancers, typically lung and gastro-intestinal cancers, suggestive of NER deficiency in defense against carcinogens from the air and food. In addition, around 20% of XP patients have neurological diseases such as neurodegeneration, dementia, and microcephaly. Mutations in any of the NER enzyme genes, i.e., XPA, XPB, XPC, XPD, XPE, XPF, XPG, plus an XPV variant (the genetic disease XPV results from mutations in a gene encoding translesional polymerase η), can cause Xeroderma pigmentosum [3,7,129,130]. These XP patients manifest clinical symptoms before an age of 2 years with the onset of cancer before the age of 10. In contrast, XP-F patients have much milder symptoms with a late onset of skin cancer [131,132]. XPF mutations are often point mutations that occasionally cause frame-shifts leading to destabilizing truncations in XPF protein [133]. XP-F patients have considerably reduced levels of both XPF and ERCC1 in fibroblasts, which results in a diminished endonuclease activity, contributing to a typically mild albeit long-lasting phenotype [132]. Some rare severe XPF deficiency cases come with neurological abnormalities [134]. The most severe case of XPF deficiency was a patient with XFE (XPF-ERCC1) progeroid syndrome. The patient was virtually completely devoid of TC-NER and GG-NER, but its symptoms, including growth retardation, neurodegeneration and accelerated aging, were mostly related to its high sensitivity for ICL-inducing agents but not to sensitivity to sun. The skin biopsy of the patient showed markedly decreased UV induced unscheduled DNA synthesis. This patient died at the age of 16. The symptoms of this patient are remarkably similar to the presentation of *Ercc1^-/-^* and *Xpf^m/m^* mouse models of accelerated aging [135]. Also, patients with ERCC1 deficiency, which appears less frequently, suffer from severe symptoms. According to Imoto et al. [136,137], an ERCC1 nonsense mutation K226X combined with a IVS6-G>A splice site mutation on the second allele, has caused Xeroderma pigmentosum with late onset of a progressive and severe neurodegeneration resulting in dementia and cortical atrophy at an age of 15. The patient died at the age of 37 [136,137].

### 3.2. Cockayne Syndrome

Cockayne syndrome (CS) is a severe disorder that causes accelerated aging. Depending on the severity CS is divided into three types. Depending on the CS type, the life expectancy can be from 3 years old to four decades [138,139]. Common symptoms are accelerated neurodegeneration, mental retardation, growth failure, profound cataracts, retinal degeneration and microcephaly, vascular abnormalities, with pneumonia as the frequent cause of death. To date, mutations in CSA/CSB, XPB, XPD, XPF and XPG genes are known to cause occasional lesions that stall replication and transcription resulting in CS due to the defect in TC-NER and HR repair mechanisms [129,140,141,142,143,144]. Even low levels of unrepaired lesions in transcriptional genes, that are not removed by other repair systems, are sufficient to shorten the life span considerably [129,130]. A rare variant of CS, XP/CS, show developmental and neurological abnormalities typical of CS, and skin and eyes phenotype typical of XP. The phenotypes of XP and CS overlap because the XPB, XPD and XPG genes belong to a group with similar characteristics, with CS having milder dermatological phenotypes than XP [140,145,146].

Recently, three cases of CS were identified which involved defects in ERCC1 or XPF. Two of the patients showed the typical CS phenotype, while the third patient had a combined XP, CS and FA phenotype (XPCS/FA). In the XPCS/FA patient the phenotype was caused by the bi-allelic XPF mutations C236R and R589W (the latter was previously also observed in XP patients) [88,135,145]. In one CS patient, the bi-allelic mutations were C236R in the SF2 helicase domain of XPF and a premature stop codon at Y577 in XPF, while the second CS patient carried a bi-allelic F231L mutation in ERCC1 which led to death in early life [145,147]. This CS patient with F231L mutation that is described by Kashiyama et al. [145] is only the second case of F231L mutation in ERCC1, and has the most severe NER deficiency. Before this F231L CS patient, another patient with F231L mutation in ERCC1 was reported by Jaspers et al. [147] to have symptoms including a heterogeneous congenital COFS and severe embryonic and postnatal growth failure. Similar to CS, some of the symptoms consisted of reduced birth weight and early microcephaly, followed by brain atrophy, hypotonia and cataracts. COFS was in the past only linked to mutations in CSB, XPD or XPG, though the phenotypes of COFS are more severe (micro-cornea with optic atrophy in COFS vs. pigmentary retinopathy in CS) [129,147,148]. The patient described by Jaspers et al. presented a new cause for COFS that is caused by a heterozygous bi-allelic mutation of ERCC1, in which in one allele Q158 was converted to a stop codon resulting in a fully dysfunctional ERCC1, while the other allele carried a C-to-G transversion causing a F231L mutation [147,149]. For the Kashiyama et al.’s patient, with the bi-allelic homozygous F231L mutation early onset of CS deficiency without an indication of COFS was observed [145]. The findings suggest that residual amounts of deficient ERCC1 could still maintain the TC-NER function at such a level that the severity, as observed for the F231L mutant patient, did not occur. Biophysical studies on F231L ERCC1-XPF dimerization domain have demonstrated the importance of the F231 residue for the stability of the ERCC1-XPF complex and thus for the activity of ERCC1-XPF [150].

### 3.3. Fanconi Anemia Disorder

This disorder was a phenotype of progressive bone marrow failure and predisposition to cancer, is associated with proteins that are involved in ICL repair. At least fifteen proteins have been identified to be associated with Fanconi anemia (FA). Recently, the sequencing of all protein-coding genes in an unclassified FA patient revealed mutations in *ERCC4* genes (encoding the XPF protein) [88]. The genetic, biochemical and functional analysis of this mutation shows that the function of XPF in ICL repair is drastically reduced without severe NER deficiency. As previously discussed for CS, another patient has also shown a combined phenotype with FA, XP and CS symptoms [145]. Summarizing, recent findings underscore a multifunctional role of ERCC1-XPF, existing beyond DNA repair. Though the frequency of diseases due to ERCC1 or XPF deficiencies is not high, it is expected that defects in ERCC1-XPF will be discovered to be associated with several other diseases.

## 4. ERCC1 and XPF Belong to the XPF Nuclease Family

The proteins ERCC1 (31 kDa) and XPF (103 kDa) (Figure 4A) do not function as individual monomers but form a heterodimeric complex. This ERCC1-XPF complex functions as a structure-specific endonuclease and is recruited to perform an incision at a ds/ss DNA junction on the 5′ side of the damage while XPG incises on 3′ side [33,151,152,153]. Both ERCC1 and XPF belong to the XPF nuclease family, also known as the XPF/MUS81 family [2,6,154,155,156]. The proteins of the XPF family are evolutionarily conserved in eukaryotes and archaea, where the members of this family exist as either heterodimers in eukaryotes or homodimers in archaea. The *N*-terminal part of XPF shows similarity to four consecutive motifs in archaeal helicases as well and though the XPF proteins have no direct orthologs in bacteria, their DNA repair mechanism are similar to the UvrABC family of bacterial NER proteins.

**Figure 5:**
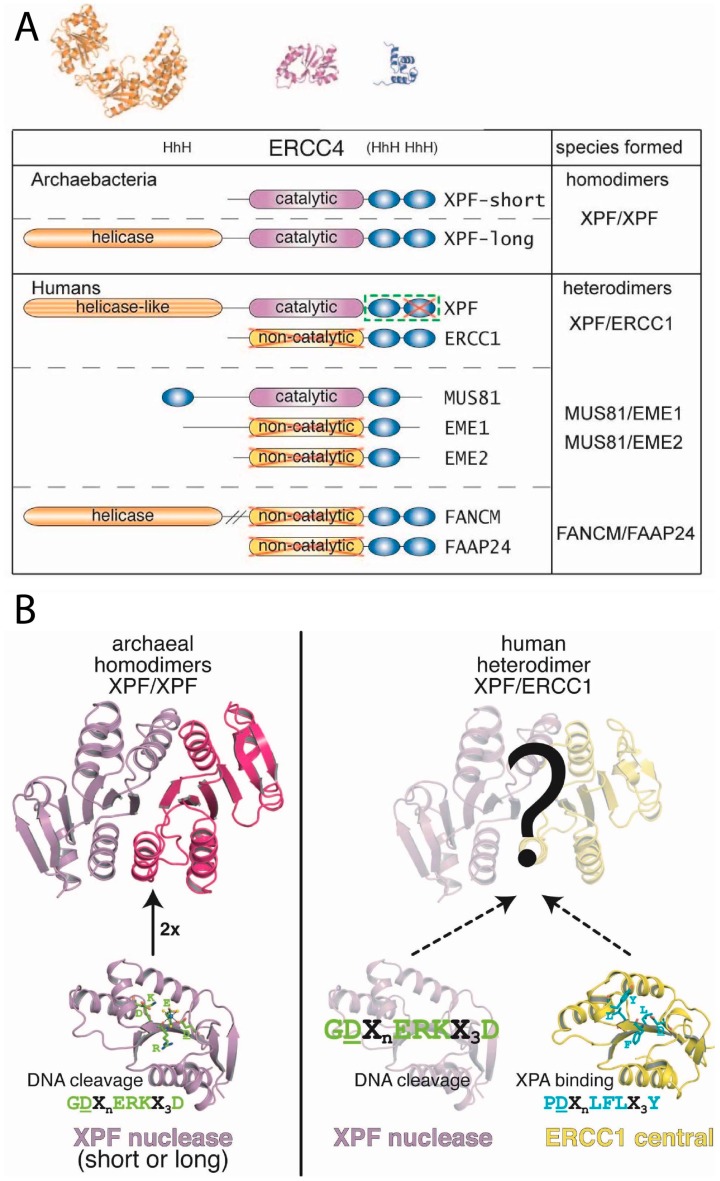
The family of XPF proteins and fold of the ERCC4 domains and corresponding function. **A**, Domain organization for the members of the family from archaea and humans, and the dimeric species formed. ERCC4 and HhH domains are indicated, while on the top the representative folds of each domain from *P. furiosus* are shown. Inactivated domains in human with respect to the original archaeal functions are red crossed. **B**, the conserved catalytic site from the archaeal nuclease is shown in stick representation, whereas the divalent cation (light blue) and the three water molecules (yellow) that assist in the octahedral coordination scheme are shown as balls. The corresponding substitutions in the human ERCC1 central domain that mediate XPA binding are shown in stick representation. The conserved signatures are indicated for each domain.

XPF consists of a *N*-terminal helicase-like domain and a central excision repair cross complementation group 4 (ERCC4) endonuclease domain, followed by a C-terminal helix-hairpin-helix (HhH)_2_ putative DNA-binding domain. The non-catalytic ERCC1 protein also contains an ERCC4-like domain—known as the central domain—that lacks the catalytic motif. Like in XPF, the central domain is followed by a helix-hairpin-helix (HhH)_2_ domain (Figure 5A) [2,6]. The dimerization of XPF and ERCC1 takes place through a direct interaction between the two (HhH)_2_ domains.

In eukaryotes, several XPF/MUS81 family members have been identified existing as a heterodimeric complex, with one catalytic and one non-catalytic subunit. An exception is FAAP24-FANCM, in which both domains are non-catalytic due to the absence of the catalytic amino-acid residues in the endonuclease domain [160,161]. In humans, there are seven homologs: XPF, ERCC1, MUS81, EME1, EME2, FANCM and FAAP24 [2,162]. These proteins have been found in four heterodimeric complexes: XPF-ERCC1, MUS81-EME1, MUS81-EME2 and FANCM-FAAP24; but also the Rad1-Rad10 (*S. cerevisiae*), Rad16-Swi10 (*S. pombe*) and HIM9-XPF (*C. elegans*) belong to this family (Figure 5) [163].

ERCC1-XPF, MUS81-EME1 and MUS81-EME2 maintain their catalytic activity through the conserved endonuclease domain in XPF and MUS81 [2,161,164]. ERCC1-XPF is involved in NER and unhooking of ICL, whereas MUS81-EME1 resolves recombination intermediates containing Holliday junctions during the G2/M phase of the cell cycle [165,166], and the MUS81-EME2 heterodimer is required for processing and restart of stalled replication forks specifically in the S-phase of the cell cycle [166]. Finally, FANCM-FAAP24 has affinity for splayed arm substrates and maybe loading FA core complex onto the chromatin during interstrand crosslink (ICL) repair [2,167,168].

In contrast to the eukaryotic members of the XPF family, several archaeal members are functional as homodimers. *P. furiosus* from euryarchaeota forms homodimers of the XPF homolog Hef (PfuHef). A shorter form of the XPF homologs found in *S. solfataricus* (SsoXPF) and *A. pernix* (ApXPF), both from crenarchaeota, lacks the helicase domain, but combines a ERCC4 nuclease domain, a (HhH)_2_ domain and a C-terminal PCNA-interaction motif (Figure 5) [2,169].

### 4.1. The Superfamily 2 Helicase-Like Domain

The helicase domains in the human XPF family members belong to the superfamily 2 (SF2) of RNA-helicases [155,170]. They show a high similarity to the *N*-terminal part of XPF in seven conserved archaeal helicase families. In human XPF, the degenerate SF2 helicase-like domain is devoid of key residues essential for ATP binding and hydrolysis activity resulting in a nonfunctional helicase domain. Although, its function remains elusive, deletion of the helicase domain from ERCC1-XPF causes a strong decrease in in vitro DNA cleavage activity [171]. The disrupted regions in the human SF2 helicase-like domain are localized in two motifs. The first is the MgATP/MgADP interacting GKT sequence, a consensus nucleotide binding motif in RNA helicases. The second motif contains acidic residues within the DEAD/DEAH box within the α-β domain, which is essential for chelating Mg^2+^ in RNA helicases. Functional SF2 helicases such as in FANCM contain a DEAH helicase domain with ATP-dependent DNA translocase and fork reversal activities, which are crucial for its role in promoting ICL repair [172,173,174,175,176]. This ATP-dependent DNA unwinding is important for the mono-ubiquitination of FA core complex and may recruit the complex to the chromatin [168]. In the archaeal homodimer, Hef, the two characteristic helicase motifs are separated by an α-helical domain that are critical for ATP dependent DNA unwinding, and contribute to branched DNA recognition [2]. The interaction of XPF with SLX4 is mediated via its helicase domain as demonstrated by mutational and deletion analysis [99]. In this way SLX4 supports both the recruitment of XPF to the ICL and its unhooking incision function [99]. This also explains recent data of a patient who had mutations in the helicase-like and nuclease domains of XPF and who was found to be deficient in both NER and ICL repair [177].

### 4.2. The ERCC4 Endonuclease Domain

The middle domain of the XPF family proteins is a metal-dependent endonuclease domain, designated as the excision repair cross complementation group 4 (ERCC4) domain. The architecture of Hef, the archaeal homolog of this domain is remarkably similar to that of the nuclease domain of type II restriction endonucleases. These contain a small endonuclease subunit (ca. 18 kDa) with a conserved structural fold common in all restriction enzymes. This subunit is composed of five- or six-stranded β-sheets, flanked on both sides by α-helices. Despite the structural homology between Hef and type II restriction enzymes, sequence homology is mostly limited to the active site residues of Hef. The homologous regions include the ERKX_3_D signature motif with an extension GDX_n_ (GDX_n_ERKX_3_D) [178]. This corresponds to the PDX_n_(E/D)XK motif of type II restriction enzymes (Figure 5B) [179]. Mutational studies indicated metal binding and/or catalytic function for these residues, while no role was found in DNA binding [162]. Similarly, as found for type II restriction endonucleases, the conserved acidic residues within the ERCC4 domain motif participate in Mg^2+^- and Mn^2+^-ion binding [162]. The polar residues in this motif including the lysine form hydrogen bonds with coordinated water molecules and activate the water molecule for nucleophilic attack in the endonuclease reaction. The hydrolysis results in breakage of the bond between a phosphate group and the 3′-oxygen of DNA [74,162,179]. During this reaction, the divalent metal ion stabilizes the departing groups [180].

### 4.3. The ERCC1 Central Domain of ERCC1

Although the overall fold of the ERCC1 central domain (cERCC1) is similar to that of the nuclease domains of archaeal species, it lacks the characteristic catalytic residues [171]. The non-catalytic domain has a completely distinct function in DNA substrate recognition. Previous NMR studies have shown that cERCC1 can interact with two highly conserved motifs in XPA (rich in glycine and glutamate) [35]. The binding site was mapped to residues 92–119 of cERCC1, the region that corresponds to the catalytic ‘signature’ of XPF nuclease domain. However, in cERCC1, the catalytic residues ″ERK″ are replaced by ″LFL″ in human ERCC1 or ″LF/YL″ in other eukaryotic ERCC1 proteins. It was also noted that cERCC1 can bind ssDNA directly. Chemical shift perturbations mapped the ssDNA-binding site to residues N99, I102, L132, K213, A214 and Q134, which is located at a hydrophobic cleft on an opposite site of cERCC1 as compared to the XPA-binding site [35,171,181]. It has been suggested that cERCC1 plays an important role in the positioning of ERCC1-XPF heterodimer near the cleavage site [35].

In the archaeal homolog of ERCC1-XPF, the nuclease domains are directly interacting to each other. However, stable interactions between the nuclease domain of human XPF and the central domain of ERCC1 have not been observed [35].

### 4.4. The Tandem Helix-Hairpin-Helix Domain (HhH)_2_

The C-terminal part of the proteins of the XPF family contains a conserved domain engaged in binding DNA junctions. The domain consists of a tandem Helix-hairpin-Helix (HhH)_2_ motif. The HhH motif is a characteristic DNA recognition motif, known to bind nonspecifically to the phosphate backbone of DNA. It can be found in proteins such as DNA and RNA polymerases, DNA ligases, DNA glycosylases and in the NER repair nuclease UvrC [156,182]. Two copies of this HhH motif are connected by an α-helix (helix γ) and together this (HhH)_2_ motif forms a globular fold. The key residues of the HhH motifs are found at positions 3, 6, 8, 9, 10, 14, 17 and 18 [159]. Of these, residues 3, 17, and 18 are responsible for hydrophobic packing of the motif, and residues 6 and 9 define a type II β-turn. Generally, on positions 8 and 10 two glycine residues are found that are also important for the β-hairpin conformation (Figure 4B). Finally, position 14 of the HhH motif is often occupied by a small hydrophobic residue such as alanine to prevent a steric clash with the first helix of the HhH motif [159,182]. This GXG sequence in the β-hairpins h_1_ and h_2_ is essential for interaction with the minor groove of duplex DNA and binds the phosphate backbone of both DNA strands separated by three base pairs. The hydrophobic residue X within the hairpin is most commonly a hydrophobic Ile, Val or Leu residue.

As mentioned before, proteins of the XPF family can form heterodimers. For this, the (HhH)_2_ domains of each protomer associate together into a stable heterodimeric endonuclease. Thus, this domain is required both for DNA binding and for dimerization in XPF family of proteins. The archaeal XPF homolog of ERCC1-XPF forms a symmetric homodimeric complex and its structure has been determined [183] Structurally this complex shows considerable similarities to that of its eukaryotic relatives. The eukaryotic heterodimers are not symmetric, and it was found for ERCC1-XPF that each of the two monomers has distinct functional roles [184]. While the ERCC1 monomer contains a canonical (HhH)_2_ motif, it was noted that in XPF the second hairpin of the (HhH)_2_ motif is evolved into a short turn containing only three residues. The tandem heterodimeric (HhH)_2_ domain is composed of a hydrophobic core and two pseudo-symmetric phenylalanine anchor residues that insert into a complementary cavity of their binding partner (Figure 4C,D) [164,171]. The structure of the heterodimeric complex of the ERCC1-XPF (HhH)_2_ domains has been determined independently by NMR [164] (Figure 4C), and by protein-crystallography [171]. For its complex formation two conserved phenylalanines, namely F293 of ERCC1 and F894 of XPF, play a key role [185]. Taking advantage of the crucial role of these phenylalanines for the stability of the ERCC1-XPF complex, recently the cavity containing the F293 sidechain has been targeted by small molecules, which led to identifying molecules that could potentially induce loss of interaction between the two proteins [186]. Such molecular induced loss-of-interaction could potentially be used to block NER and has been suggested for additional use during chemotherapy to promote apoptosis in tumor cells by accumulation of damage [187].

The dimerization of the two (HhH)_2_ domains implies that there exist two surfaces for potential DNA binding in ERCC1-XPF. It has been observed that for the ERCC1 and XPF archaeal homologs of this family (the archaeal XPF homodimer) the (HhH)_2_ domain engages in both upstream and downstream duplex regions of their branched DNA substrates (Figure 3B). DNA-binding properties of the heterodimeric ERCC1-XPF complex have been studied before. Tripsianes et al. [164,171] showed that the ERCC1 (HhH)_2_ domain can interact with dsDNA through both canonical h_1_ and h_2_ β-hairpin motifs [164]. Later, Das et al. [184] showed that the XPF (HhH)_2_ domain, which in vitro can form stable homodimers, has a larger interaction interface compared to the heterodimer and lacks the second canonical β-hairpin motif that is essential for dsDNA-binding. The DNA binding studies revealed that XPF homodimer can bind single-strand DNA (ssDNA). Together, this led to a model for binding of ERCC1-XPF at a junction between dsDNA and ssDNA, as present near the DNA damage site [164,184].

## 5. Perspectives

Understanding DNA repair pathways such as NER, DSB, SSB and ICL repair is of basic interest for understanding fundamental cellular processes. It also forms the basis for understanding molecular details of diseases when defects occur in these pathways. Additional insight will help us to find relations between the different DNA repair pathways and to define relations to other cellular processes such as ERCC1 ubiquitination. Interaction studies have shown multiple stances of the involvement of ERCC1 and XPF in several DNA repair pathways. Detailed knowledge of these interactions can unravel the function of ERCC1 and XPF in these pathways and how this function is regulated. It forms a foundation for understanding the molecular basis for several related diseases and could lead to therapeutic strategies to overcome those [104,150,188]. Moreover, the involvement of ERCC1-XPF in the removal of damages induced during platinum-chemotherapy and radiotherapy of various cancer types, results in tumor resistance to these treatments. This reflects another horizon in medical importance of ERCC1-XPF, and has increased the attention to possible regulation of ERCC1-XPF expression and activity [17,189]. Therefore, the correlation between ERCC1 level and the resistance to cancer treatments makes this complex a potential biomarker for the prediction of patient survival in several cancer types [186,190,191].
